# Septin-3 autoimmunity in patients with paraneoplastic cerebellar ataxia

**DOI:** 10.1186/s12974-023-02718-9

**Published:** 2023-03-30

**Authors:** Ramona Miske, Madeleine Scharf, Kathrin Borowski, Nicole Rieckhoff, Bianca Teegen, Yvonne Denno, Christian Probst, Kersten Guthke, Ieva Didrihsone, Brigitte Wildemann, Klemens Ruprecht, Lars Komorowski, Sven Jarius

**Affiliations:** 1 Institute for Experimental Immunology, affiliated to EUROIMMUN AG, Lübeck, Germany; 2 Clinical Immunological Laboratory Prof. Dr. med. Winfried Stöcker, Lübeck, Germany; 3 Department of Neurology, Städtisches Klinikum Görlitz, Görlitz, Germany; 4 Department of Neurology, Hermann-Josef-Krankenhaus, Erkelenz, Germany; 5 Molecular Neuroimmunology Group, Department of Neurology, University of Heidelberg, Heidelberg, Germany; 6 Department of Neurology, Charité - Universitätsmedizin Berlin, corporate member of Freie Universität Berlin and Humboldt-Universität zu Berlin, Berlin, Germany

**Keywords:** Autoimmune cerebellar ataxia, Paraneoplastic neurological syndrome, Autoantibodies, Septin-3, Melanoma, Small-cell lung cancer, Septins, Septin-5, Septin-6, Septin-7, Septin-11, Immunoglobulin G, Autoimmune encephalitis, Cerebellitis, Cerebellum, Autoimmunity, Immunoprecipitation, Paraneoplastic cerebellar degeneration

## Abstract

**Background:**

Septins are cytoskeletal proteins with filament forming capabilities, which have multiple roles during cell division, cellular polarization, morphogenesis, and membrane trafficking. Autoantibodies against septin-5 are associated with non-paraneoplastic cerebellar ataxia, and autoantibodies against septin-7 with encephalopathy with prominent neuropsychiatric features. Here, we report on newly identified autoantibodies against septin-3 in patients with paraneoplastic cerebellar ataxia. We also propose a strategy for anti-septin autoantibody determination.

**Methods:**

Sera from three patients producing similar immunofluorescence staining patterns on cerebellar and hippocampal sections were subjected to immunoprecipitation followed by mass spectrometry. The identified candidate antigens, all of which were septins, were expressed recombinantly in HEK293 cells either individually, as complexes, or combinations missing individual septins, for use in recombinant cell-based indirect immunofluorescence assays (RC-IIFA). Specificity for septin-3 was further confirmed by tissue IIFA neutralization experiments. Finally, tumor tissue sections were analyzed immunohistochemically for septin-3 expression.

**Results:**

Immunoprecipitation with rat cerebellum lysate revealed septin-3, -5, -6, -7, and -11 as candidate target antigens. Sera of all three patients reacted with recombinant cells co-expressing septin-3/5/6/7/11, while none of 149 healthy control sera was similarly reactive. In RC-IIFAs the patient sera recognized only cells expressing septin-3, individually and in complexes. Incubation of patient sera with five different septin combinations, each missing one of the five septins, confirmed the autoantibodies’ specificity for septin-3. The tissue IIFA reactivity of patient serum was abolished by pre-incubation with HEK293 cell lysates overexpressing the septin-3/5/6/7/11 complex or septin-3 alone, but not with HEK293 cell lysates overexpressing septin-5 as control. All three patients had cancers (2 × melanoma, 1 × small cell lung cancer), presented with progressive cerebellar syndromes, and responded poorly to immunotherapy. Expression of septin-3 was demonstrated in resected tumor tissue available from one patient.

**Conclusions:**

Septin-3 is a novel autoantibody target in patients with paraneoplastic cerebellar syndromes. Based on our findings, RC-IIFA with HEK293 cells expressing the septin-3/5/6/7/11 complex may serve as a screening tool to investigate anti-septin autoantibodies in serological samples with a characteristic staining pattern on neuronal tissue sections. Autoantibodies against individual septins can then be confirmed by RC-IIFA expressing single septins.

**Supplementary Information:**

The online version contains supplementary material available at 10.1186/s12974-023-02718-9.

## Introduction

Paraneoplastic neurological syndromes (PNS) are rare autoimmune diseases of the nervous system associated with cancer outside the brain. Sera of patients with PNS often contain autoantibodies targeting neuronal proteins. Diagnostically, these autoantibodies can function as markers for distinct neurological autoimmune diseases as well as for the underlying tumors [1, 2].

Septin proteins as target antigens in neurological autoimmune diseases were first described in patients with cerebellar ataxia and anti-septin-5 autoantibodies [3, 4]. Recently, anti-septin-7 autoantibodies were identified in patients with encephalopathy and myelopathy [5], suggesting that autoantibodies targeting different septins may be associated with distinct neurological phenotypes.

Septins belong to a large conserved family of guanosine triphosphate (GTP)-binding proteins widely expressed in all metazoan tissues. In humans, at least 13 different septins exist. They are divided into four groups according to sequence similarities: septin-2 group (septins-1,-2,-4, and -5), septin-3 group (septins-3,-9, and -12), septin-6 group (septins-6,-8,-10,-11, and -14) and septin-7 [6]. In vivo, septins self-assemble into hetero-oligomers that contain septins from three or four different groups [6]. These core particles are the building blocks for higher order structures such as filaments, rings and coils, which function in a variety of cellular processes including cell division, cellular polarization, morphogenesis, and membrane trafficking. In the nervous system, septins play a role in neurite formation, as well as pre- and post-synaptic signaling processes, including neurotransmitter exocytosis.

Here, we report on septin-3 as a novel autoimmune target antigen in patients with paraneoplastic cerebellar ataxia.

## Methods

Reagents were obtained from Merck, Darmstadt, Germany, or Sigma-Aldrich, Heidelberg, Germany, if not specified otherwise.

### Patients

Serum samples from the three septin-3 IgG-positive patients were sent in for routine autoantibody testing for diagnostic purposes, including identification of antibody targets. One of the samples (PS1) was previously found to be positive for low-titer GABAB receptor and GAD65 antibodies [7]. All patients gave written informed consent. Anonymized sera of 149 healthy blood donors, 59 patients with multiple sclerosis (ethics committee of Charité—Universitätsmedizin Berlin, EA4/231/20 and EA4/018/17) and 52 patients with anti-neuronal antibodies (leftover material, laboratory Prof. Stöcker, Lübeck, Germany) were used as controls.

### Indirect immunofluorescence assays

Indirect immunofluorescence assays (IIFAs) were performed using microscopy slides mounted with a biochip array consisting of brain tissue cryosections (rat hippocampus, rat or primate cerebellum, murine encephalon), recombinant HEK293 cells separately expressing brain antigens (Hu, Yo, Ri, CV2, PNMA2, ITPR1, Homer 3, CARP VIII, ARHGAP26, ZIC4, DNER/Tr, GAD65, GAD67, amphiphysin, recoverin, GABAB receptor, glycine receptor, DPPX, IgLON5, NMDA receptor, AMPA receptor, mGluR1 receptor, mGluR5 receptor, GLURD2 receptor, LGI1, CASPR2, M1-AQP4, M23-AQP4, MOG, ATP1A3, NCDN), recombinant acetone-fixed HEK293 cells expressing either different histidine-tagged septin combinations (septin-3/5/6/7/11; septin-5/6/7/11; septin-3/6/7/11; septin-3/5/7/11; septin-3/5/6/11; septin-3/5/6/7) or septin-3, -5, -6, -7, or -11 separately, and non-transfected HEK293 cells as control substrate. Each biochip mosaic was incubated with 70 µL of PBS-diluted serum or CSF at room temperature for 30 min, washed with PBS-Tween, and immersed in PBS-Tween for 5 min. In a second step, either Alexa Fluor 488 labelled goat anti-human IgG (Jackson Research, Suffolk, United Kingdom), fluorescein isothiocyanate (FITC)-labelled goat anti-human IgG (EUROIMMUN Medizinische Labordiagnostika AG, Lübeck, Germany) or IgG subclass specific FITC-labelled mouse anti-human IgG (1–4, Sigma-Aldrich) were applied and incubated at room temperature for 30 min. If required, nucleic acid stain (TO-PRO™-3 Iodide, Fisher Scientific, Waltham, USA) was added during the second incubation step. Slides were washed again with a flush of PBS-Tween and then immersed in PBS-Tween for 5 min. Slides were embedded in PBS-buffered, DABCO-containing glycerol (approximately 20 µL per field) and examined by fluorescence microscopy. Samples were classified as positive or negative based on fluorescence intensity of the transfected cells in direct comparison with non-transfected cells and control samples. Endpoint titers refer to the highest dilution showing visible fluorescence.

For neutralization assays, diluted serum samples were pre-incubated with HEK293 cell extracts containing the overexpressed recombinant antigen or with empty vector-transfected HEK293 control extracts 1 h prior to tissue incubation.

Results were evaluated by two independent observers using a EUROStar II microscope (EUROIMMUN Medizinische Labordiagnostika AG, Lübeck, Germany) or an LSM700 (Zeiss, Jena, Germany).

### Immunoprecipitation

Cerebellum from rat was dissected and shock-frozen in liquid nitrogen. The tissues were homogenized in solubilization buffer (100 mmol/L tris–HCl pH 7.4, 150 mmol/L sodium chloride, 2.5 mmol/L ethylenediaminetetraacetic acid, 0.5% (w/v) sodium deoxycholate, 1% (w/v) Triton X-100) containing protease inhibitors (Complete mini, Roche Diagnostics, Penzberg, Germany) at 4 °C. Insoluble material was sedimented by centrifugation at 21,000×*g* at 4 °C for 15 min. For immunoprecipitation, 500 µL of the supernatant were incubated at 4 °C overnight with 15 µL of patient sera and then with Protein G Dynabeads (ThermoFisher Scientific, Dreieich, Germany) for another 3 h to capture immunocomplexes. Beads were washed 3 times with PBS, and eluted with NuPage LDS sample buffer (ThermoFisher Scientific, Schwerte, Germany) containing 25 mmol/L dithiothreitol at 70 °C for 10 min. Carbamidomethylation with 59 mM iodoacetamide (Bio-Rad, Hamburg, Germany) was performed prior to SDS-PAGE (NuPAGE, ThermoFisher Scientific, Schwerte, Germany). Separated proteins were visualized with Coomassie Brilliant Blue (G-250) (Merck), and identified by mass spectrometric analysis as described elsewhere [8].

### Recombinant expression of septin proteins in HEK293 cells

The cDNA encoding human septin-3, septin-5, septin-6, septin-7 and septin-11 was obtained from Source BioScience UK Limited (clones IRCMp5012E0331D, IRAUp969E0781D, IRAUp969G0159D, IRCMp5012B107D and IRATp970F0181D).

The coding sequences were amplified by PCR using the template cDNA and DNA oligonucleotide primers (Additional file 6: Table S1). The amplification products were digested with Esp3I or with NcoI/XhoI as indicated and ligated with NcoI/XhoI linearized pTriEx-1 (Merck, Darmstadt, Germany).

The septin proteins were transiently expressed in the human cell line HEK293 following PEI-mediated transfection (Exgene 500), according to the manufacturer’s instructions (Biomol GmbH, Hamburg, Germany). For IIFA, cells were grown on cover slides and acetone-fixed 2 days after transfection. For the production of recombinant cell lysates, cells were harvested 5 days after transfection and lysed by shear-stress. The lysates were stored in aliquots at − 80 °C until further use. His-tagged septin-3 was enriched from HEK293 cells expressing septin-3-His in addition to non-tagged septin-5, -6, -7, and -11 by immobilized metal ion affinity chromatography combined with anion exchange chromatography.

### Tumor tissue staining for septin-3

Formalin-fixed paraffin-embedded melanoma and lymph node metastasis tissue from patient 1 were sectioned (4 μm). As positive control, mouse cerebellum tissue was sectioned as well. Slices were placed onto slides, deparaffinized, rehydrated, and subjected to heat-induced epitope-retrieval using Target Retrieval Solution (pH 9, 3-in-1, Dako, Hamburg, Germany) according to the supplier’s instructions. Subsequently, the slides were washed with Tris-buffered saline (TBS) containing 0.05% Tween-20 at room temperature. Blocking was performed with serum-free protein block (Thermo Fisher Scientific, Schwerte, Germany) for 10 min. Polyclonal rabbit anti-septin-3 (HPA003548, Sigma-Aldrich, Taufkirchen, Germany) was diluted 1:500 or 1:1,000 in Dako antibody diluent and then applied for 30 min. Polyclonal rabbit anti-septin-11 (HPA003459, Sigma-Aldrich) and monoclonal rabbit anti-GABARB2 receptor (ab75838, Abcam) were used as controls in a 1:75 and 1:250 dilution, respectively. As a negative control, rabbit immunoglobulin fraction (X0936, Dako, Hamburg, Germany) was used. Envision + HRP Rabbit detection system (Dako, Inc., Santa Clara, US) was used according to manufacturer’s instructions to detect bound rabbit IgG. This system is based on a horseradish peroxidase-labelled, avidin/biotin-free polymer conjugated with the secondary antibody to reduce background staining from endogenous peroxidase and pseudoperoxidase; 3,3′-diaminobenzidine (DAB +) chromogen is used for visualization. Hematoxylin (Leica Biosystems, Wetzlar, Germany) was used for counterstaining. Slides were mounted with the water-free mounting medium Neo-Mount (VWR, Darmstadt, Germany).

## Results

### Clinical and paraclinical features

Clinical and paraclinical findings, tumor associations, therapy and outcome of the three patients are summarized in Table 1. All patients were male and ≥ 60 years of age at onset of neurological symptoms (64, 69, and 60 years, respectively). All presented with a subacute progressive cerebellar syndrome, including dysarthria and gait ataxia. One patient had prominent downbeat nystagmus. Two patients had metastatic malignant melanoma, detected 5 and 19 months before onset of neurological symptoms, and one patient had metastatic small cell lung cancer, detected 3 months after onset of neurological symptoms. An MRI scan performed 9 months after onset of ataxia revealed mild cerebellar atrophy in patient 2, while for the other two patients cerebral MRIs were normal 3 and 17 months (patient 1) or 2 months (patient 3) after neurological onset. CSF findings indicated mild pleocytosis in 2 patients and total protein elevation in all 3. Importantly, all three patients showed evidence of an intrathecal IgG production, consistent with an autoimmune process. Immunotherapies comprised pulsed high-dose intravenous methylprednisolone in all patients and plasma exchange, intravenous immunoglobulins and rituximab in one patient; they were associated with no further progression, or transient improvement, but no sustained improvement of neurological symptoms (Table 1). One patient died at home with the direct cause of his death being unknown.

**Table 1:** Clinical and paraclinical findings, tumor associations, therapies, and outcome of three patients with septin-3 IgG antibodies

Patient #, sex, age at neurological onset	Clinical summary	Cancer	Cancer therapy (start before/after onset of neurological symptoms)	Septin-3 IgG	Coexisting antibodies	Brain MRI	CSF findings	Immunotherapy (response)	Follow-up time (months)	Outcome
#1 male, 64^a^	Subacute progressive cerebellar syndrome with dysarthria, bilateral limb and gait ataxia	Malignant melanoma (Clark level IV) right groin with inguinal lymph node metastases	Adjuvant immunotherapy with interferon-alpha-2b (5 months before)	Serum 1:32.000	Antinuclear Abs (1:320), GAD 65 Abs (28 units/ml, reference range 10 units/ml), GABAB receptor Abs 1:100	Few small unspecific microangiopathic frontal lesions	3 cells/µl, TP^b^ 718 mg/l, QAlb mildly elevated, intrathecal IgG synthesis (56%)^c^	3 × 500 mg IVMP (none), PE (transient), IVIG (transient), rituximab (transient)	29	Death
#2 male, 69	Subacute progressive cerebellar syndrome with dysarthria and marked gait ataxia	Malignant melanoma (Clark level IV) back with lung, liver and axillary lymph node metastases	Irradiation; adjuvant immunotherapy with nivolumab; dabrafenib and trametinib (19 months before)	Serum 1:10.000, CSF 1:1000	None	Mild cerebellar atrophy, moderate microangiopathy	13 cells/µl, TP^b^ 611 mg/l, normal QAlb, CSF-specific OCB	5 × 1000 mg IVMP (partial improvement)	32	Cerebellar syndrome stable, but worsening (epileptic seizures) due to cerebral metastases
#3 male, 60	Subacute progressive cerebellar syndrome with dysarthria, ataxia left leg, down beat nystagmus	Small-cell lung cancer with mediastinal lymph node metastases	Cisplatin/etoposide and atezolizumab (3 months after)	Serum 1:100.000	Antinuclear Abs (1:160)	Normal	20 cells/µl, TP^b^ 889 mg/l, CSF-specific OCB	5 × 250 mg IVMP, oral prednisolone (no further worsening)	15	Neurological symptoms stable

Abs, antibodies; CSF, cerebrospinal fluid; IgG, immunoglobulin G; IVMP, intravenous methylprednisolone; IgG, immunoglobulin G; IVIG, intravenous immunoglobulins; MRI, magnetic resonance imaging; OCB, oligoclonal bands; PE, plasma exchange; TP, total protein

^a^Patient 1 was previously reported in [7]

^b^Upper limit of normal of CSF total protein: 450 mg/l

^c^Normal value for intrathecal IgG production: 0%

### Patient sera show a similar pattern in indirect immunofluorescence assays with neuronal tissues

**Fig. 1:**
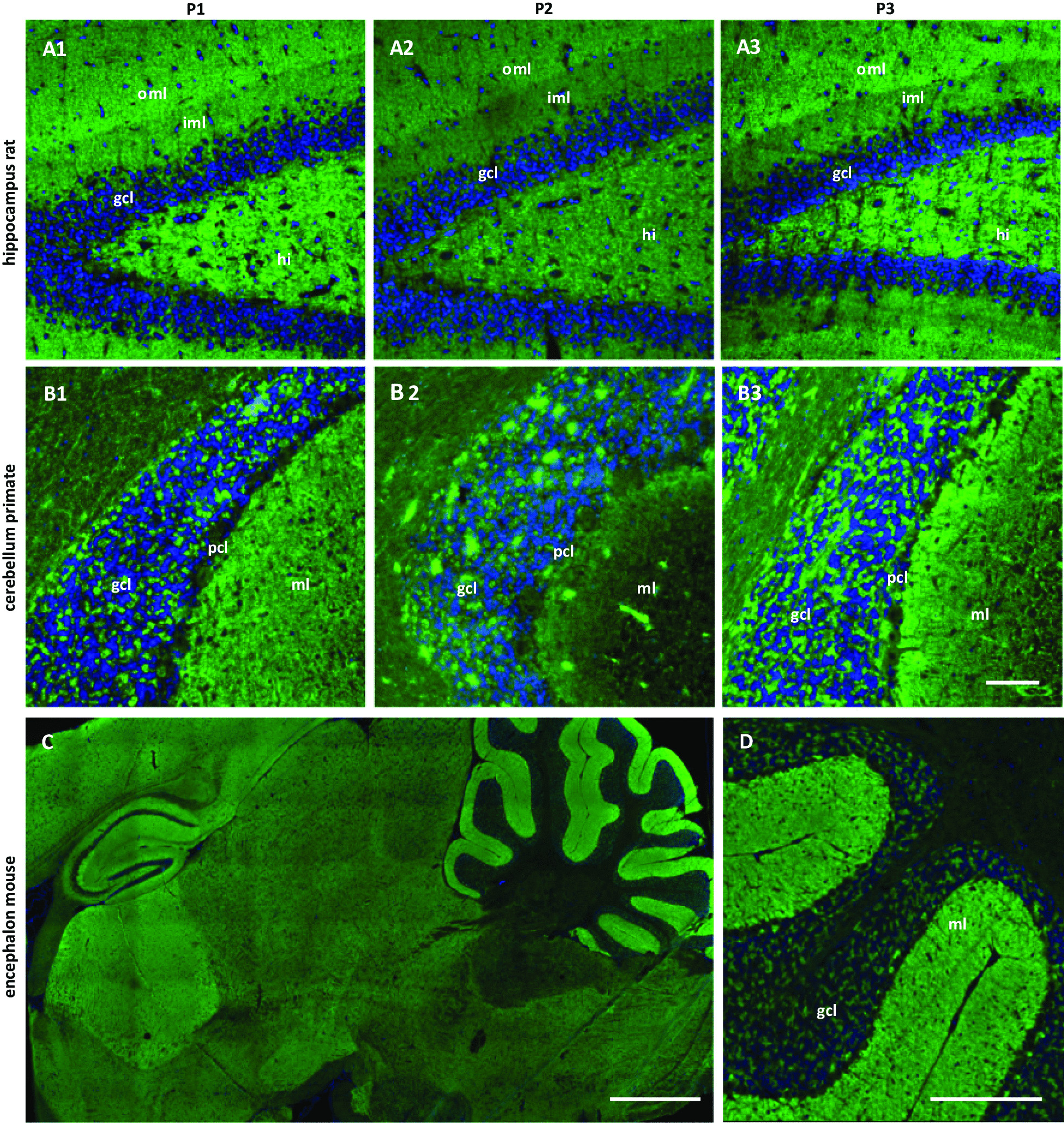
Immunofluorescence staining of central nervous tissues with patient sera. Cryosections of rat hippocampus, primate cerebellum and murine encephalon were incubated with patient sera (**A**, **B** Patients 1–3, 1:100; **C**, **D** Patient 1, 1:100) in the first step, and with Alexa Fluor 488 labeled goat anti-human IgG in the second step (green). A granular staining of the molecular layer (ml) was obtained on hippocampus and cerebellum (**A**, **B**). Additionally, a blotchy fluorescence of the granular layer was observed on cerebellum (**B**). On hippocampus, staining of the outer molecular layer (oml) is more intense compared to the inner molecular layer (iml) (**A**). On murine encephalon (**C**), the strongest reactivity was detected in the molecular layer of the cerebellum (**D**). Nuclei were counterstained by incubation with TO-PRO-3 iodide or DAPI (blue). (Scale bar: **A**, **B** 100 µm, **C** 1000 µm, **D** 250 µm.)

In tissue IIFA, sera of the three patients revealed a characteristic, similar staining pattern on rat hippocampus, with more intense staining of the outer than the inner molecular layer (Fig. 1A). Furthermore, the sera stained the granular layer and molecular layer of rat and primate cerebellum, while Purkinje cells were mostly spared (Fig. 1B). On murine encephalon sections, the strongest reactivity was detected in the molecular layer of the cerebellum (Fig. 1C, D). Interestingly, the characteristic staining pattern on rat hippocampus was likewise observed with anti-septin-5 or anti-septin-7 positive control sera (Additional file 1: Fig. S1), suggesting that the three patient sera might also contain anti-septin autoantibodies. Serum endpoint titers in IIFA (as detected using rat hippocampus sections) ranged between 1:1000 and 1:10,000.

Testing of the three patient sera using IIFA with recombinant HEK293 cells separately expressing 30 different neuronal antigens (Hu, Yo, Ri, CV2, PNMA2, ITPR1, Homer 3, CARP VIII, ARHGAP26, ZIC4, DNER/Tr, GAD65, GAD67, amphiphysin, recoverin, GABAB receptor, glycine receptor, DPPX, IgLON5, NMDA receptor, AMPA receptor, mGluR1 receptor, mGluR5 receptor, GLURD2 receptor, LGI1, CASPR2, M1-AQP4, M23-AQP4, MOG, ATP1A3, NCDN) revealed no positive results except for serum of patient 1, which showed a positive reaction with GABAB receptor-transfected HEK293 cells, as previously reported [7], with an endpoint titer of 1:100. At the same time, co-existing anti-glutamate decarboxylase antibodies were detected at low levels by ELISA (28 units/ml, upper reference limit 10 units/ml) in serum of patient 1. However, because of the particularly strong brain tissue IIFA pattern caused by this patient’s serum (endpoint titer of 1:1000 on rat hippocampus at Laboratory Stöcker, Lübeck; 1:20,000 on cerebellum at the University of Heidelberg using different methodology [7]) the presence of an additional autoantibody was suspected.

### Patient sera immunoprecipitate septin-3, -5, -6, -7 and -11

**Fig. 2:**
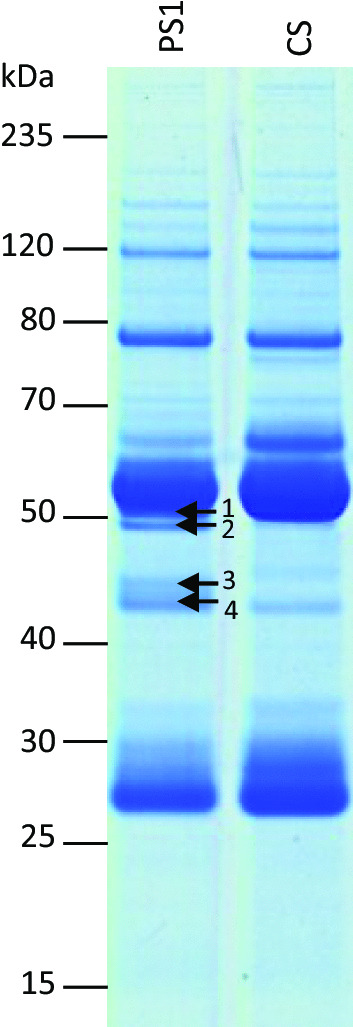
Immunoprecipitation and antigen identification with patient serum. **A** SDS-PAGE of the immunoprecipitates of patient serum 1 (PS1) or control serum (CS) with cerebellar lysates stained with colloidal Coomassie revealed four specific bands (1–4) between 40 and 50 kDa in the eluate fraction of PS1 but not CS. Mass spectrometry analysis identified the following proteins: **band 1** septin-6,-7,-11; **band 2** septin-6,-7,-11; **band** **3** septin-3,-5; **band 4** septin-3,-5 in the eluate fraction of PS1

In immunoprecipitates of all three patient sera and cerebellar lysates, four bands were revealed by SDS-PAGE/Coomassie staining in the 35–55 kDa range (Fig. 2). Mass spectrometry identified five members of the septin family (septin-3, -5, -6, -7 and -11) in gel fragments picked at the position of these bands.

### Recombinant septin-3, -5, -6, -7 and -11 build complexes in HEK293 cells

Immobilized metal chelate affinity chromatography purification of recombinant septin-3-His together with non-tagged septin-5, -6, -7, and -11 in HEK293 cells was performed to prove that these five septin proteins form a complex. By mass spectrometry analysis, all transfected septins were detected clearly, indicating that septin heterocomplexes have been formed in the recombinant HEK293 cells (Additional file 2: Fig. S2).

### Patient sera recognize septin-3 in recombinant indirect immunofluorescence assays

**Fig. 3:**
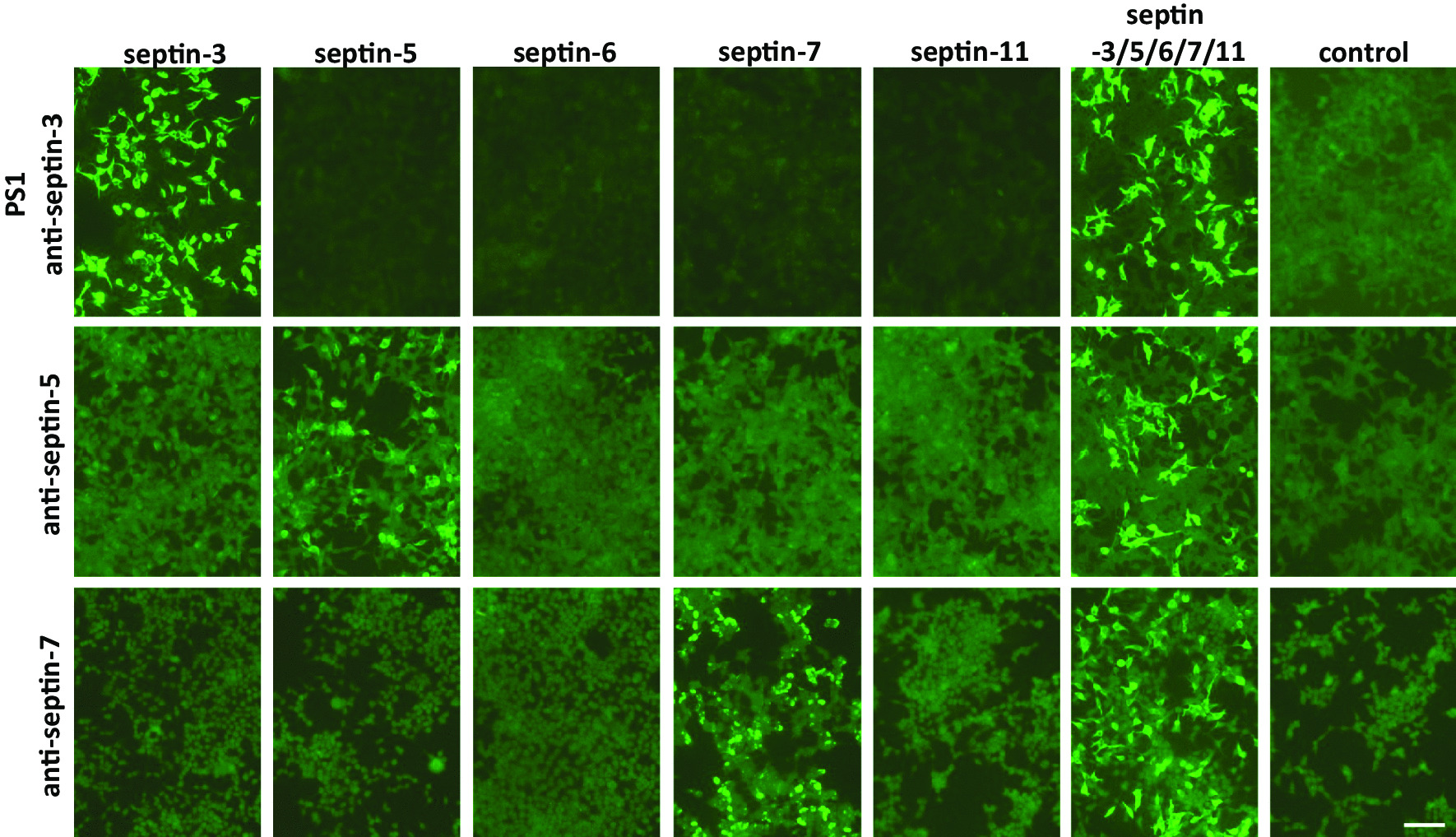
Indirect immunofluorescence analysis of single septin or septin-3/5/6/7/11 complex transfected HEK293 cells with patient serum. Acetone-fixed recombinant HEK293 cells expressing septin-3, septin-5, septin-6, septin-7 or septin-11 individually or co-expressing septin-3/5/6/7/11 or an empty vector-transfected control were incubated with the patient serum (PS1) or an anti-septin-5 or anti-septin-7 positive serum (1:100). All anti-septin positive sera showed a positive reaction with the recombinant septin-3/5/6/7/11 expressing HEK293 cells. Additionally, PS1 recognized cells expressing recombinant septin-3 but none of the other single septin expressing HEK293 cells. Recombinant septin-5 or septin-7 expressing HEK293 cells were recognized by the anti-septin-5 or anti-septin-7 positive control serum, respectively. (Scale bar 100 µm.)

As the exact epitope of the patients’ antibodies was unclear, septin-3, -5, -6, -7 and -11 were expressed either individually or together in HEK239 cells and tested using RC-IIFA. All three patient sera showed a positive reaction with septin-3 expressing HEK293 cells (IgG end titers 1:32,000, 1:10,000 and 1:100,000; no IgA/IgM) and the HEK293 cells expressing the septin-3/5/6/7/11 complex (IgG end titers 1:32,000, 1:10,000 and 1:100,000; no IgA/IgM) (Fig. 3). The serum anti-septin-3 autoantibodies belonged to the IgG1 and IgG2 subclass (patient 1: IgG1 and IgG2; patient 2: IgG2; patient 3: IgG2 > IgG1). CSF was available from patient 2 and reacted positively with the HEK293-septin-3 cells and the HEK293-septin-3/5/6/7/11 cells (IgG titer 1:1,000 with each substrate; IgG2 subclass). As no blood–CSF barrier dysfunction was present in this patient (as indicated by a normal CSF/serum albumin quotient), intrathecal synthesis of septin-3 IgG is likely based on a CSF/serum ratio of 1:10 (compared to a normal mean CSF/serum ratio for total IgG of ~ 1:300). In contrast, sera with known anti-septin-5- or anti-septin-7-reactivity, respectively, used as controls, reacted with the septin-5-transfected cells (end titer 1:1,000) or septin-7-transfected cells (end titer 1:3200), respectively, and with the septin-3/5/6/7/11 complex cells (end titer 1:3200 and 1:1000, respectively) but not with the septin-3-expressing HEK293 cells (Fig. 3).

Experiments with HEK293 cells expressing different combinations of only four of the five septins confirmed anti-septin-3 specificity of the three patient sera. Indeed, the combination of septin-5/6/7/11 missing septin-3 was the only one not recognized by the patient sera (Additional file 3: Fig. S3).

None of 149 healthy control sera showed a positive reaction with the HEK293-septin-3/5/6/7/11 complex cells. However, four of 149 healthy control sera were positive in RC-IIFA with HEK239 cells expressing recombinant septin-3 in a 1:1000 dilution (end titer CS7, CS24, CS74 1:1,000; CS74 1:3,200), but importantly, none of these sera showed the characteristic staining pattern in tissue IIFA at a dilution of 1:10 and 1:100 (data not shown), suggesting that the antibodies present in the four controls were different from those in the three patients. This was confirmed by immunoprecipitation experiments, in which none of the tissue IIFA-negative control sera that reacted with the septin-3-expressing HEK293 cells immunoprecipitated septin proteins from hippocampal lysates (Additional file 4: Fig. S4).

Therefore, we suggest that the septin-3/5/6/7/11 complex should be used as a first-line tool for the work-up of samples suspected to contain anti-septin-3 autoantibodies because of the tissue pattern in IIFA.

Furthermore, 59 sera of patients with multiple sclerosis and 50 sera of patients positive for different anti-neuronal autoantibodies (10 × anti-Yo, 10 × anti-Ri, 10 × anti-GAD65, 10 × anti-ITPR1, 10 × Sez6l2) were analyzed using RC-IIFA with HEK293-septin-3/5/6/7/11 complex cells as controls. Only 1/59 sera of patients with multiple sclerosis and 1/50 sera of patients with anti-neuronal autoantibodies reacted positive in RC-IIFA with HEK293-septin-3/5/6/7/11 complex cells at a titer of 1:320 in both cases. However, similar to the RC-IIFA-positive healthy controls, none of these two control sera showed the distinct staining pattern observed with the three patient sera in the tissue IIFA (data not shown).

### Tissue reactivity of patient sera is caused by anti-septin-3 autoantibodies

**Fig. 4:**
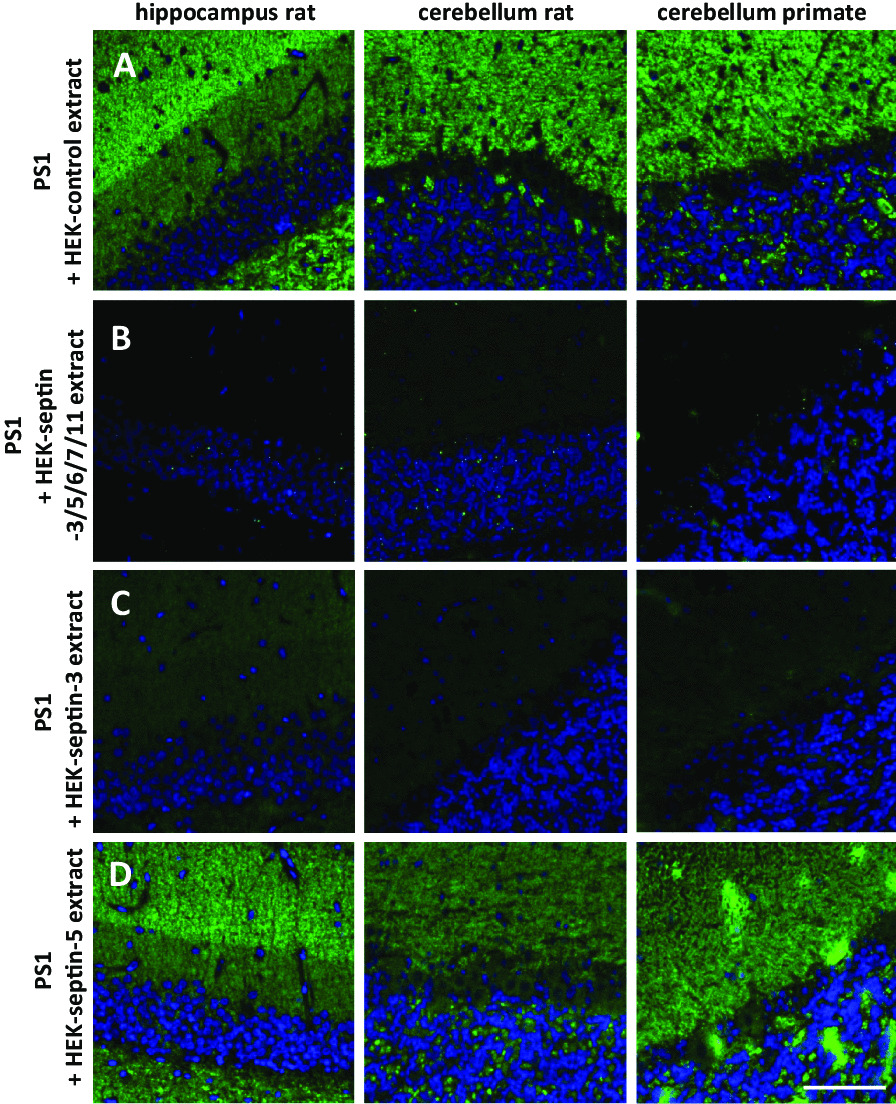
Neutralization of indirect immunofluorescence reaction on neuronal tissues with patient serum. Patient serum 1 (PS1, 1:100) was pre-incubated with extracts of HEK293 cells transfected with empty control vector (**A**) or with septin-3/5/6/7/11 (**B**), septin-3 (**C**) or septin-5 (**D**) before an indirect immunofluorescence assay with neuronal cryosections and Alexa Fluor 488 labeled goat anti-human IgG as secondary antibody was performed. The extract containing the septin-3/5/6/7/11 complex or septin-3 alone (**B**, **C**) abolished the immune reaction of PS1 on rat hippocampus, rat and primate cerebellum. The HEK293 control and the HEK293-septin-5 extracts (**A**, **D**) had no effect. Nuclei were counterstained with TO-PRO-3 iodide (blue). (Scale bar: 100 µm.)

To determine whether the tissue IIFA pattern was caused by anti-septin-3 autoantibodies, we performed neutralization assays. Pre-incubation of patient serum with HEK293-septin-3 or HEK293-septin-3/5/6/7/11 extract abolished the tissue reactivity of serum of patient 1, while incubation with empty vector-transfected HEK293 cell extract had no effect (Fig. 4). By contrast, pre-incubation with the HEK293-septin-5 extract had no effect on the tissue reactivity of the anti-septin-3-positive patient serum (Fig. 4), but clearly reduced the reactivity of an anti-septin-5-positive serum (Additional file 5: Fig. S5).

### Septin-3 is expressed in patient tumor tissue

**Fig. 5:**
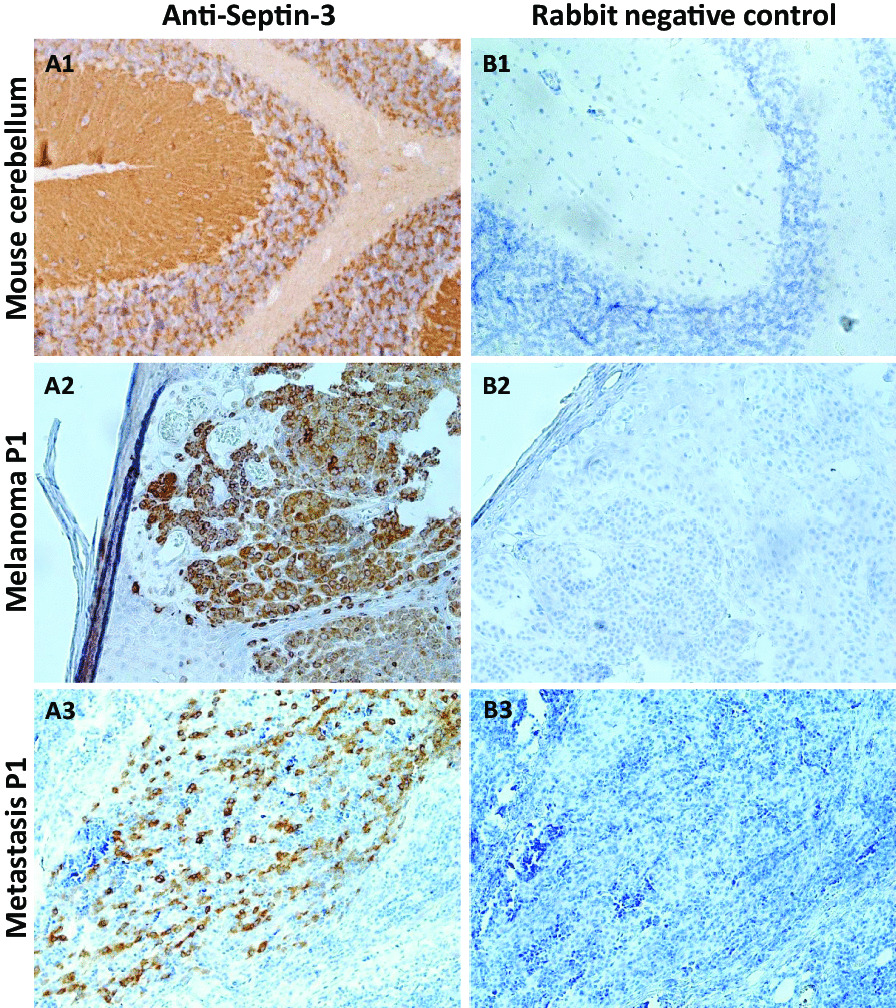
Expression of septin-3 in tumor specimens obtained from patient 1. Immunohistochemical staining of mouse cerebellum (**A1**, **B1**) and patient’s cancer specimens (**A2-3**, **B2-3**) incubated with anti-septin-3 rabbit commercial antibody diluted 1:1000 (**A1**) or 1:500 (**A2-3**) and control rabbit serum (**B1-3**). (**A2**, **B2**) Melanoma, patient 1; (**A3**, **B3**) lymph node metastasis, patient 1. (Magnification: 200-fold.)

Tumor tissue was only available from patient 1. The analysis of paraffin-embedded tumor sections from patient 1 using conventional IHC with an anti-septin-3-specific commercial antibody, revealed areas with high expression of septin-3 both in the melanoma and in the lymph node metastasis (Fig. 5).

IHC of mouse cerebellum sections with the anti-septin-3 antibody showed staining of the granular layer and molecular layer (Fig. 5) similar to the pattern observed with patient sera in IIFA with rat cerebellum (Fig. 1). In contrast, IHC using a commercial anti-septin-11 antibody or a commercial anti-GABAB2 receptor antibody showed no staining of the tumor tissues of patient 1, though antibody functionality was demonstrated by IHC with mouse neuronal tissue sections (data not shown).

## Discussion

In this study we identified septin-3 as a new target antigen in autoimmune cerebellar ataxia. All three patients analyzed had cancer (2 × melanoma, with a lymph node metastasis in one of them; 1 × small cell lung cancer with lymph node metastases), suggesting that septin-3 IgG-associated autoimmune cerebellar ataxia may be a novel paraneoplastic neurological syndrome (PNS). Indeed, septin-3, which is usually absent outside the CNS [9, 10], was ectopically and strongly expressed in two tumor samples from one of our patients, corroborating this notion. However, as no tumor material was available of patient 2 and 3, we could not investigate septin-3 expression in these cases.

Human septin-3 is expressed at high level in the human cerebellum, the cerebral cortex, including the temporal cortex, and in the hippocampus [9, 11]. In the neocortex, it was found in association with neuropil and punctate structures suggestive of synaptic junctions [11]. For human septin-3, a role in synaptic vesicle recycling [10], synaptogenesis and neuronal development has been suggested [11]. A role of septins in tumorigenesis has been discussed previously, as septin mutations or changes in expression levels have been observed in a variety of cancers [12–14]. Mutations of septin-3 have most frequently been observed in lung, skin and intestinal tumors [12].

Previously, autoantibodies against another neuron-specific septin, septin-5, have been reported in patients with cerebellar ataxia, which was associated with eye movement disorders in most of them. However, no associated tumor was found in any of the published cases associated with anti-septin-5 autoantibodies [3–5].

Most recently, we and others identified antibodies against septin-7, which is ubiquitously expressed throughout the body. The clinical phenotypes of septin-7 IgG-positive patients were more diverse [5]. The majority developed encephalopathy, partially accompanied by neuropsychiatric symptoms. A tumor was detected in 4/15 anti-septin-7-positive patients.

Importantly, in the present study none of the septin-3-positive patient sera cross-reacted with septin-5 or septin-7, as demonstrated by RC-IIFA. In line with this finding, neither the serum nor the CSF of a septin-3-positive patient (patient #1 in the present study) reacted significantly with isolated recombinant septin-5, septin-7, septin-1, septin-2, septin-4 (transcript variants 1 and 3), septin-6 (transcript variant II and V), septin-9, septin-11 or septin-12 included in a microarray assay independently performed at the University of Heidelberg (data not shown; see ref. [15] for methodology); recombinant septin-3 was not available at that time and thus not included in this commercial protein array by the manufacturer.

Altogether, the different clinical phenotypes reported in patients with anti-septin-3, anti-septin-5 or anti-septin-7 autoimmunity so far suggest that these autoantibodies may be markers for different clinical presentations.

Eight out of ten anti-septin-5 or -7 patients with immunotherapy data available improved after treatment. [3–5]. In the present study, only transient improvement (patient 1 and 2) or stabilization of symptoms but no improvement (patient 3) was observed in the anti-septin-3-positive patients. However, little is known about how different treatments and tumor associations influence short-term and long-term outcome.

Although septins are reported as intracellular proteins, a pathogenic role of anti-septin-5 and -7 autoantibodies was suggested because these autoantibodies react in IIFA with living hippocampal neurons and had electrophysiological effects on cortical neurons [5]. However, the pathogenic mechanism of autoantibodies targeting intracellular septin proteins is not understood so far and requires further studies. It is currently unknown whether septin-3 can reach the cell surface during exocytosis, which would make it accessible to extracellular IgG.

Notably, the onset of cerebellar ataxia in patient 1 and patient 2 was preceded by tumor therapy with interferon-alpha (INF-alpha-2b) or the checkpoint inhibitor nivolumab, respectively. Induction of autoimmune diseases has been previously described as a relatively frequent side effect of interferon-alpha treatment [16–18]. In a clinical trial, production of autoantibodies (including anticardiolipin, antithyroglobulin, and antinuclear antibodies) was observed in as much as 52% of patients with malignant melanoma treated with pegylated IFN-alpha-2b [19]. It thus appears conceivable that IFN-alpha treatment may have contributed to the development of autoimmunity in patient 1. Similarly, a substantial proportion of cancer patients treated with immune checkpoint inhibitors (ICI) develop immune-related adverse events (irAE), with a reported incidence of ~ 20% of high grade irAE for anti-PD1 treatment and of ~ 60% for the combination of anti-PD1 and anti-CTLA4 [20]. Neurological manifestations with grade 3 or 4, including autoimmune encephalitis, were reported with an incidence of 1.5% after ICI treatment [21]. Melanomas are very rare among the tumors usually associated with PNS [22]. However, ICI could induce such complications also in patients with tumors not commonly associated with paraneoplastic neurological syndromes [23, 24]. In particular, Sechi et al. reported that among 63 patients with ICI-related neurologic autoimmunity 27 had melanoma [23]. In patient 3, PD1-inhibition was only initiated 3 months after onset of neurological symptoms.

In serum of patient 1, co-existing antibodies to GABAB receptor (titer RC-IIFA 1:100) and low-titer antibodies to GAD65 (28 units/ml, reference range 10 units/ml; negative pancreas tissue IIFA) were detected in serum but not in CSF [7] in addition to septin-3 antibodies (end titer RC-IIFA 1:32.000), which is in line with a more widespread effect of interferon-alpha on (auto)antibody production. While the tissue IIFA neutralization experiments indicate that the tissue pattern was caused by anti-septin-3 autoantibodies (Fig. 4), it cannot be ruled out that the patient’s clinical symptoms were in part also related to autoimmunity to GABAB receptor and/or GAD65.

Patient sera applied in this work precipitated a septin-3/5/6/7/11 complex from rat cerebellar lysates. Tsang et al. described septin complexes including these five septins which contain one member of each septin group and septin-6 and -11 from the same group in rat hippocampal neurons and suggested a functional role of these complexes in neurotransmitter release [26]. The immunoprecipitation of a complex with two septins from the same group might indicate that septin filaments bound by the patients’ autoantibodies contain two distinct septin octamers. In addition, Fujishima et al. demonstrated that in mature nerve terminals of mice, septin-3 directly binds to septin-5 and septin-7, which form a heteromeric complex [27]. RC-IIFA with HEK293-cells expressing septin-3,-5,-6,-7 or -11 separately demonstrated that our patients’ autoantibodies target septin-3 but none of the other septin proteins, indicating that the other septin proteins were co-immunoprecipitated. Complex formation of recombinant septin-3, -5, -6, -7, and -11 was demonstrated in this work by co-purification of septin-5, -6, -7, and -11 with septin-3-His from HEK293 cells (Additional file 2: Fig. S2).

The observation that the anti-septin-3-positive patient sera described in this study did not bind recombinant septin-5,-6,-7 or -11 suggests that the epitopes recognized by anti-septin-3 autoantibodies are located in the heterogeneous N- or C-terminal region of septin-3, rather than the conserved central GTPase and polybasic domains [6].

Control sera reacted more frequently with the HEK293-septin-3 RC-IIFA substrate compared to the HEK293-septin-3/5/6/7/11 complex-expressing cells. Several studies indicate that single septins that are missing their partners become unstable and aggregate into amyloid-like structures [28, 29]. It is therefore conceivable that septin heterocomplexes are the dominant physiological species. Testing only with cells overexpressing septin-3 alone, as done for RC-IIFA, might give false-positive results; as such overexpression might create unphysiological filaments exposing neoepitopes prone to antibody binding.

As a strategy for the detection of anti-septin-3 autoantibodies in patient samples with a characteristic pattern using IIFA with hippocampal or cerebellar tissue sections, we would thus recommend an IIFA with HEK293 cells expressing the recombinant septin-3/5/6/7/11 complex as screening assay. Positive samples should then be analyzed further in an RC-IIFA with single septin expressing HEK293-cells including HEK293-septin-3 cells to confirm specificity for the individual septin. In contrast, positive results obtained only by use of septin-3-transfected HEK293-based assays and without confirmation in tissue-based IIFA and heterocomplex-based assays should be regarded with caution and may be non-specific.

Septin-3 antibodies form part of a broader spectrum of novel autoantibodies associated with cerebellar ataxia that have been discovered over the past two decades (e.g., [3–5, 15, 30–40]), some of which are of paraneoplastic origin. Revealing an underlying autoimmune pathogenesis and/or paraneoplastic etiology in patients presenting with cerebellar ataxia of unknown cause may substantially help to guide treatment decisions and hopefully lead to better outcomes in the future.

## Conclusions

Together, our data indicate that autoantibodies against the septin-heterocomplex-integrated form of septin-3 may represent a novel biomarker in a paraneoplastic form of autoimmune cerebellar ataxia. After septin-5 and septin-7, septin-3 is the third member of the septin protein family which is linked to neuronal autoimmunity and many undetected cases might possibly exist. Antibodies against all three septins can be detected by RC-IIFA using HEK293 cells expressing septin-3/5/6/7/11. Thus we suggest inclusion of the septin-3/5/6/7/11 complex in neuronal autoimmunity routine testing.

## Supplementary Information

**Additional file 1: Figure S1.** Immunofluorescence staining of rat hippocampus with different anti-septin-positive sera. Cryosections of rat hippocampus were incubated with patient serum 1 (PS1, anti-septin-3 positive), an anti-septin-5-positive control serum, and an anti-septin-7-positive control serum (1:100), respectively, in the first step, and with Alexa Fluor 488 labeled goat anti-human IgG in the second step (green). A more intense staining of the outer molecular layer (oml) compared to the inner molecular layer (iml) was observed with these three different anti-septin positive sera. Nuclei were counterstained by incubation with TO-PRO-3 iodide (blue). (Scale bar: 100 µm).

**Additional file 2: Figure S2.** Purification of septin-3/-5/-6/-7/-11 complex by immobilized metal ion affinity chromatography (IMAC). His-tagged septin-3 and non-tagged septin-5, -6, -7, and -11 were coexpressed in HEK293 cells. Septin-3-His was enriched by IMAC combined with anion exchange chromatography. The fraction was analyzed by ESI-TOF mass spectrometry and visualized by SDS-PAGE stained with Coomassie. Identified recombinant septins are shown in the table (numbers of identified peptides in parentheses).

**Additional file 3: Figure S3.** Indirect immunofluorescence analysis of HEK293 cells expressing different combinations of septin proteins with patient serum. Acetone-fixed recombinant HEK293 cells expressing the septin-3/5/6/7/11 complex or different combinations of only four of the five septins were incubated with the patient serum (PS1) or an anti-septin-5 or anti-septin-7-positive control serum (1:100). PS1 did not react with the combination lacking septin-3, but with all other combinations. In contrast, the sera comprising an autoantibody to septin-5 or septin-7 did not show a positive reaction with the combinations lacking septin-5 or septin-7, respectively. (Scale bar: 100 µm).

**Additional file 4: Figure S4.** Immunoprecipitation and antigen identification with patient serum and control sera. SDS-PAGE of the immunoprecipitates of patient serum 1 (PS1) or control sera (CS) with cerebellar lysates stained with colloidal Coomassie. Mass spectrometry analysis of the 35–55 kDa range was performed with every sample. Septins were identified above cutoff only in the immunoprecipitate of PS1 but not in any of the four control sera.

**Additional file 5: Figure S5.** Neutralization of indirect immunofluorescence reaction on neuronal tissues with anti-septin-5 positive serum. An anti-septin-5 positive serum was pre-incubated with extracts of HEK293 cells transfected with empty control vector or with septin-5 before an indirect immunofluorescence assay with neuronal cryosections and Alexa Fluor 488 labeled goat anti-human IgG as secondary antibody was performed. The extract containing overexpressed septin-5 abolished the immune reaction of the anti-septin-5 positive serum on rat hippocampus, rat and primate cerebellum. Nuclei were counterstained by incubation with TO-PRO-3 iodide (blue). Please note that vascular staining on primate tissue is not septin-3-specific but was caused by cross-reaction of the goat anti-human IgG secondary antibody with primate IgG and is thus regularly seen with this type of assay (Scale bar: 100 µm).

**Additional file 6: Table S1.** Additional information on cDNA, oligonucleotide primers, and vectors used for recombinant expression of septin proteins in HEK293 cells. cDNA, complementary deoxyribonucleic acid; HEK293, human embryonic kidney 293.

## Data Availability

All data generated or analyzed during this study are included in this published article.
