# Supplementary figures and images for: Septin-3 autoimmunity in patients with paraneoplastic cerebellar ataxia
Source: J Neuroinflammation. 2023 Mar 30;20:88. doi: 10.1186/s12974-023-02718-9 (PMC10061979; doi:10.1186/s12974-023-02718-9)

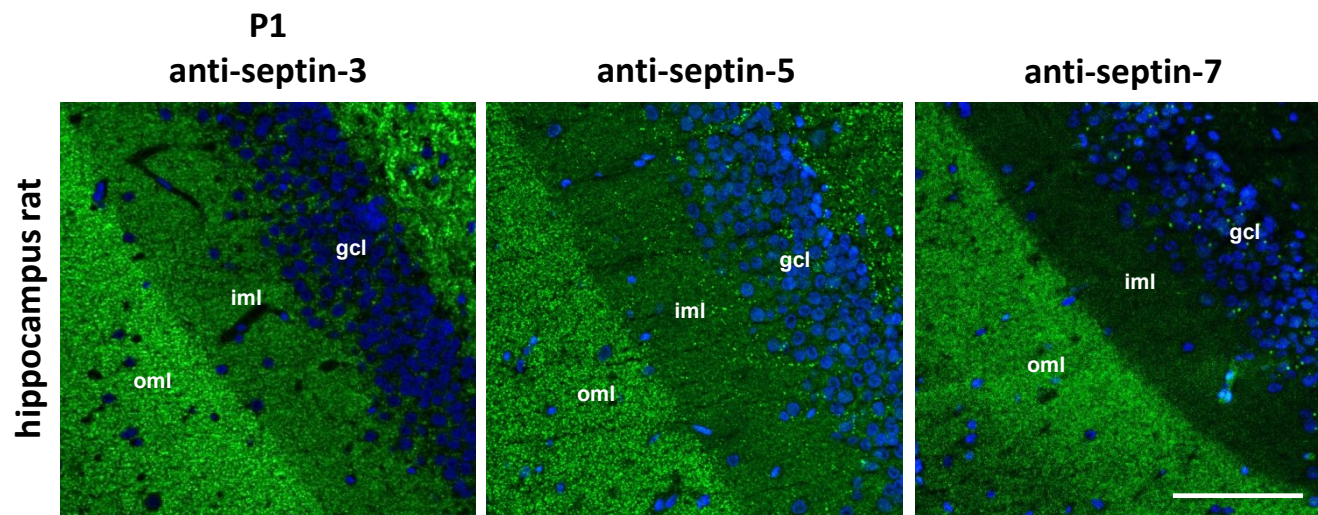

Supplement: Supplementary file 1 — Additional file 1: Figure S1. Immunofluorescence staining of rat hippocampus with different anti-septin-positive sera. Cryosections of rat hippocampus were incubated with patient serum 1 (PS1, anti-septin-3 positive), an anti-septin-5-positive control serum, and an anti-septin-7-positive control serum (1:100), respectively, in the first step, and with Alexa Fluor 488 labeled goat anti-human IgG in the second step (green). A more intense staining of the outer molecular layer (oml) compared to the inner molecular layer (iml) was observed with these three different anti-septin positive sera. Nuclei were counterstained by incubation with TO-PRO-3 iodide (blue). (Scale bar: 100 µm). [file 12974_2023_2718_MOESM1_ESM.pdf]

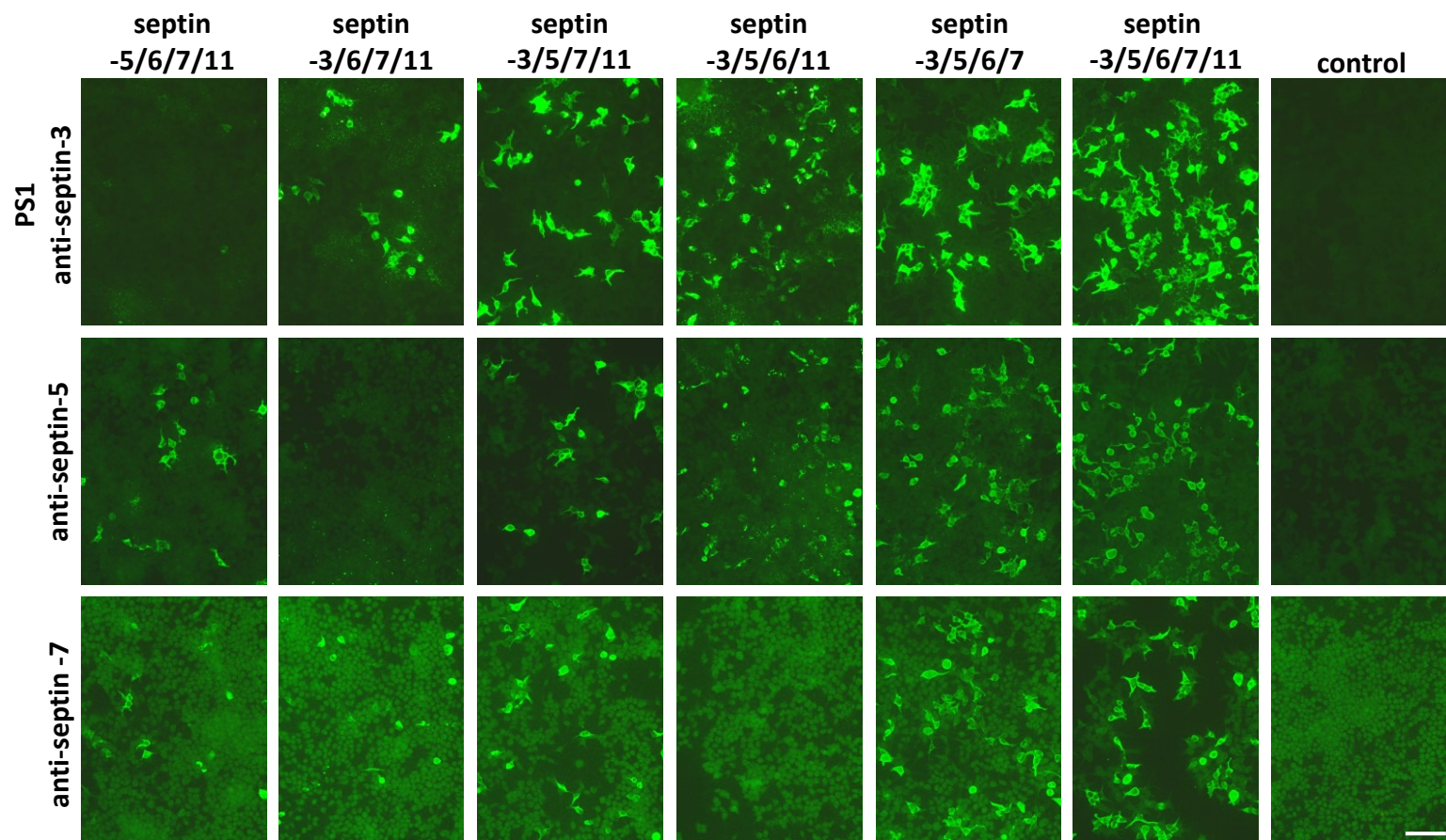

Supplement: Supplementary file 3 — Additional file 3: Figure S3. Indirect immunofluorescence analysis of HEK293 cells expressing different combinations of septin proteins with patient serum. Acetone-fixed recombinant HEK293 cells expressing the septin-3/5/6/7/11 complex or different combinations of only four of the five septins were incubated with the patient serum (PS1) or an anti-septin-5 or anti-septin-7-positive control serum (1:100). PS1 did not react with the combination lacking septin-3, but with all other combinations. In contrast, the sera comprising an autoantibody to septin-5 or septin-7 did not show a positive reaction with the combinations lacking septin-5 or septin-7, respectively. (Scale bar: 100 µm). [file 12974_2023_2718_MOESM3_ESM.pdf]

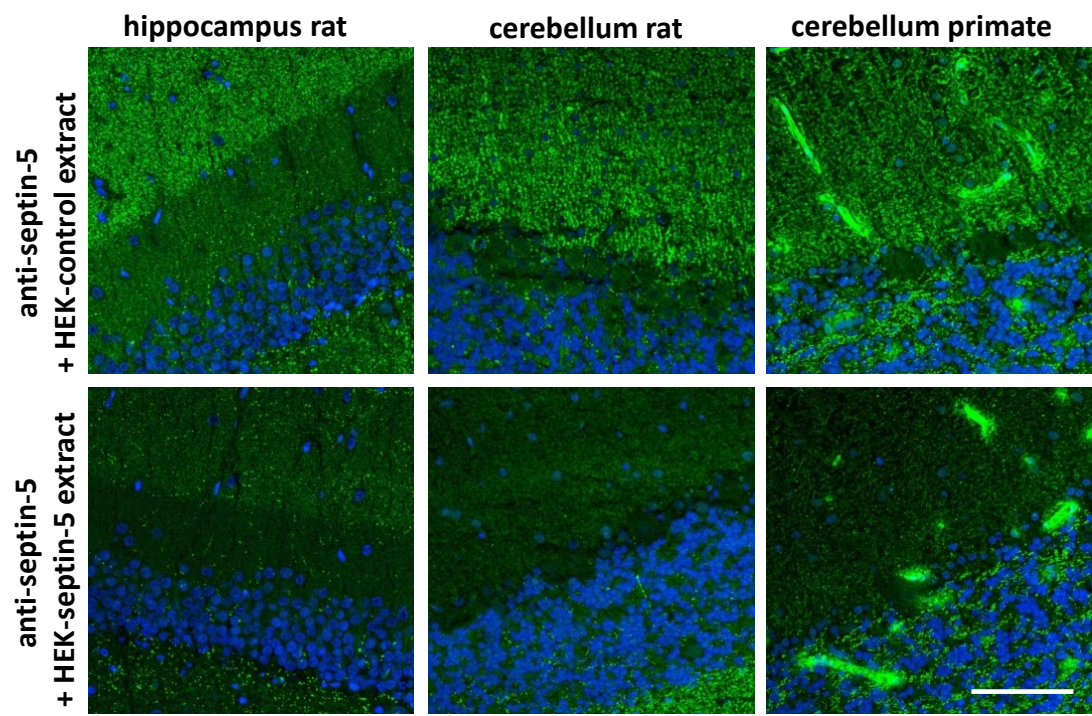

Supplement: Supplementary file 5 — Additional file 5: Figure S5. Neutralization of indirect immunofluorescence reaction on neuronal tissues with anti-septin-5 positive serum. An anti-septin-5 positive serum was pre-incubated with extracts of HEK293 cells transfected with empty control vector or with septin-5 before an indirect immunofluorescence assay with neuronal cryosections and Alexa Fluor 488 labeled goat anti-human IgG as secondary antibody was performed. The extract containing overexpressed septin-5 abolished the immune reaction of the anti-septin-5 positive serum on rat hippocampus, rat and primate cerebellum. Nuclei were counterstained by incubation with TO-PRO-3 iodide (blue). Please note that vascular staining on primate tissue is not septin-3-specific but was caused by cross-reaction of the goat anti-human IgG secondary antibody with primate IgG and is thus regularly seen with this type of assay (Scale bar: 100 µm). [file 12974_2023_2718_MOESM5_ESM.pdf]
