# Supplementary material for: Septin-3 autoimmunity in patients with paraneoplastic cerebellar ataxia
Source: J Neuroinflammation. 2023 Mar 30;20:88. doi: 10.1186/s12974-023-02718-9 (PMC10061979; doi:10.1186/s12974-023-02718-9)
Supplement: Supplementary file 2 — Additional file 2: Figure S2. Purification of septin-3/-5/-6/-7/-11 complex by immobilized metal ion affinity chromatography (IMAC). His-tagged septin-3 and non-tagged septin-5, -6, -7, and -11 were coexpressed in HEK293 cells. Septin-3-His was enriched by IMAC combined with anion exchange chromatography. The fraction was analyzed by ESI-TOF mass spectrometry and visualized by SDS-PAGE stained with Coomassie. Identified recombinant septins are shown in the table (numbers of identified peptides in parentheses). [file 12974_2023_2718_MOESM2_ESM.pdf]

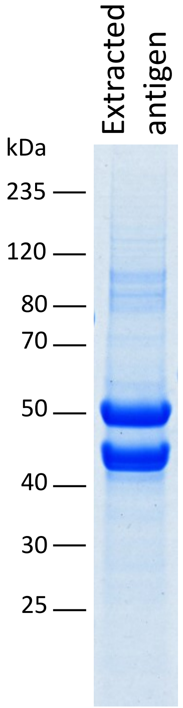

Sample	Analysis	Mass spectrometry analysis (number of identified peptides)
HEK293-SEPT3-His + SEPT5 + SEPT6 + SEPT7 + SEPT11	Whole sample	Septin-7 (56)
		Septin-3-His (49)
		Septin-5 (49)
		Septin-6 (50)
		Septin-11 (51)
