# Supplementary material for: Septin-3 autoimmunity in patients with paraneoplastic cerebellar ataxia
Source: J Neuroinflammation. 2023 Mar 30;20:88. doi: 10.1186/s12974-023-02718-9 (PMC10061979; doi:10.1186/s12974-023-02718-9)
Supplement: Supplementary file 4 — Additional file 4: Figure S4. Immunoprecipitation and antigen identification with patient serum and control sera. SDS-PAGE of the immunoprecipitates of patient serum 1 (PS1) or control sera (CS) with cerebellar lysates stained with colloidal Coomassie. Mass spectrometry analysis of the 35–55 kDa range was performed with every sample. Septins were identified above cutoff only in the immunoprecipitate of PS1 but not in any of the four control sera. [file 12974_2023_2718_MOESM4_ESM.pdf]

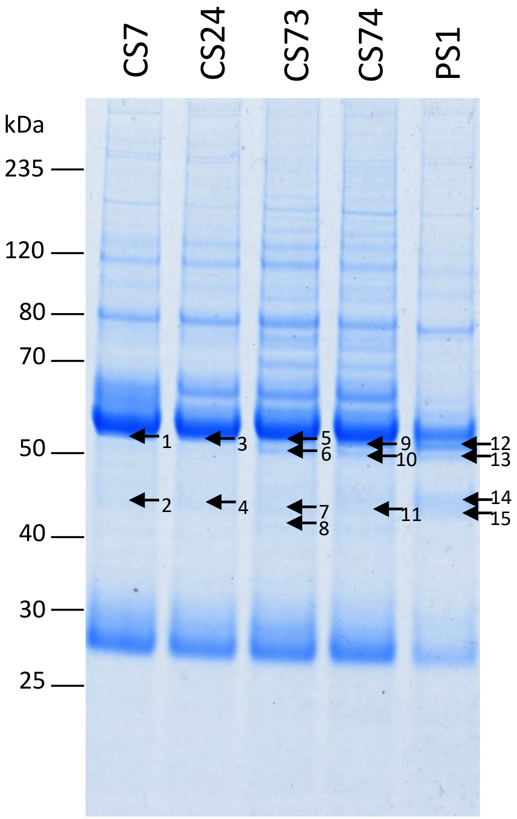

Sample	Band	Mass spectrometry analysis (number of identified peptides)
CS_7	1	ATP synthase subunit alpha, mitochondrial (7)*
	2	Actin (11)*
CS_24	3	Calcium/calmodulin-dependent protein kinase type II subunit alpha (7)*
	4	Actin (11)*
CS_73	5	Fibrinogen gamma chain *Homo sapiens* (15), ATP synthase subunit alpha, mitochondrial (10)*
	6	Fibrinogen gamma chain *Homo sapiens* (27),<br>Lipoamide acyltransferase component of branched-chain alpha-keto acid dehydrogenase complex, mitochondrial (17)*
	7	Complement C3 *Homo sapiens* (15), Haptoglobin *Homo sapiens* (13), Actin (11)*
	8	Complement C3 *Homo sapiens* (17), Haptoglobin *Homo sapiens* (12)*
CS_74	9	ATP synthase subunit alpha, mitochondrial (18), Fibrinogen gamma chain *Homo sapiens* (13)*
	10	Fibrinogen gamma chain *Homo sapiens* (29)*
	11	Complement C3 *Homo sapiens* (18), Glyceraldehyde-3-phosphate dehydrogenase (13)*
PS1	12	Septin-11 (28), Septin-6 (22), Septin-7 (19)
	13	Septin-7 (24), Septin-11 (17), Septin-6 (14)
	14	Septin-5 (15), Septin-3 (17)
	15	Septin-3 (17), Septin-5 (15)

* Above cut off (>3 peptides) no septins identified
