# Supplementary material for: Septin-3 autoimmunity in patients with paraneoplastic cerebellar ataxia
Source: J Neuroinflammation. 2023 Mar 30;20:88. doi: 10.1186/s12974-023-02718-9 (PMC10061979; doi:10.1186/s12974-023-02718-9)
Supplement: Supplementary file 6 — Additional file 6: Table S1. Additional information on cDNA, oligonucleotide primers, and vectors used for recombinant expression of septin proteins in HEK293 cells. cDNA, complementary deoxyribonucleic acid; HEK293, human embryonic kidney 293. [file 12974_2023_2718_MOESM6_ESM.docx]

**Suppl. Table 1. Additional information on cDNA, oligonucleotide primers, and vectors used for recombinant expression of septin proteins in HEK293 cells.** Abbreviations: cDNA, complementary deoxyribonucleic acid; HEK293, human embryonic kidney 293.

| **Gene** | **cDNA** | **Cleavage site** | **Oligonucleotide from 5´ to 3´ end** | **Vector** |
| --- | --- | --- | --- | --- |
| SEPTIN3 | IRCMp5012E0331D,  Source BioScience | Esp3I | atacgtctcacatgtccaaagggctcccagagac | pTriEx-1 |
|  |  |  | AAACGTCTCATCGAGTTCAGCAGTGGGGCAGGGGGTGGCT |  |
| SEPTIN5 | IRAUp969E0781D,  Source BioScience | Esp3I | atacgtctcacatgagcacaggcctgcggtacaagag | pTriEx-1 |
|  |  |  | AAACGTCTCATCGAGCTGGTCCTGCATCTGCTGC |  |
| SEPTIN6 | IRAUp969G0159D, Source BioScience | Esp3I | atacgtctcacatggcagcgaccgatatagctcgc | pTriEx-1 |
|  |  |  | AAACGTCTCATCGAGTTCAGTACACAGCCATGGATTA |  |
| SEPTIN7 | IRCMp5012B107D,  Source BioScience | Esp3I | atacgtctcccatgtcggtcagtgcgagatccgctgctgctgagga | pTriEx-1 |
|  |  |  | ATACGTCTCCTCGAGttaAAAGATCTTCCCTTTCTTCTTGTTC |  |
| SEPTIN11 | IRATp970F0181D,  Source BioScience | NcoI | ataccatggccgtggccgtggggagaccg | pTriEx-1 |
|  |  | XhoI | TATCTCGAGTTATGTGAAGCTTGCATTTTTCTTATCC |  |
